# Transient Protein-Protein Interaction of the SH3-Peptide Complex via Closely Located Multiple Binding Sites

**DOI:** 10.1371/journal.pone.0032804

**Published:** 2012-03-22

**Authors:** Seungsoo Hahn, Dongsup Kim

**Affiliations:** Department of Bio and Brain Engineering, Korea Advanced Institute of Technology (KAIST), Daejeon, South Korea; University Of Oxford, United Kingdom

## Abstract

Protein-protein interactions play an essential role in cellular processes. Certain proteins form stable complexes with their partner proteins, whereas others function by forming transient complexes. The conventional protein-protein interaction model describes an interaction between two proteins under the assumption that a protein binds to its partner protein through a single binding site. In this study, we improved the conventional interaction model by developing a Multiple-Site (MS) model in which a protein binds to its partner protein through closely located multiple binding sites on a surface of the partner protein by transiently docking at each binding site with individual binding free energies. To test this model, we used the protein-protein interaction mediated by Src homology 3 (SH3) domains. SH3 domains recognize their partners via a weak, transient interaction and are therefore promiscuous in nature. Because the MS model requires large amounts of data compared with the conventional interaction model, we used experimental data from the positionally addressable syntheses of peptides on cellulose membranes (SPOT-synthesis) technique. From the analysis of the experimental data, individual binding free energies for each binding site of peptides were extracted. A comparison of the individual binding free energies from the analysis with those from atomistic force fields gave a correlation coefficient of 0.66. Furthermore, application of the MS model to 10 SH3 domains lowers the prediction error by up to 9% compared with the conventional interaction model. This improvement in prediction originates from a more realistic description of complex formation than the conventional interaction model. The results suggested that, in many cases, SH3 domains increased the protein complex population through multiple binding sites of their partner proteins. Our study indicates that the consideration of general complex formation is important for the accurate description of protein complex formation, and especially for those of weak or transient protein complexes.

## Introduction

Protein-protein interactions are essential in virtually every process within cells. The rate of protein complex formation is governed by diffusion and geometric constraints, followed by a structural reorganization to form a stable complex [1], [2]. For certain proteins, a transient complex, the “encounter complex”, accelerates the formation of the protein complex [3]. The encounter complex is primarily formed from charge-charge interactions between proteins and operates by reducing the conformational search space [4]. The existence of the encounter complex has been verified by several kinetic experiments [5] and visualized using NMR paramagnetic relaxation enhancement, which is used for relatively weak and fast-exchanging protein-protein complexes [6]. Protein complexes that are bound by non-covalent interactions are in dynamic equilibrium (i.e., they continuously switch between free and bound states) [5], [7]. If a peptide ligand has multiple binding sites that are located close to one another, an encounter complex would increase the speed in such a way that a protein shuttles between each binding site in the peptide ligand [7].

**Figure 1:**
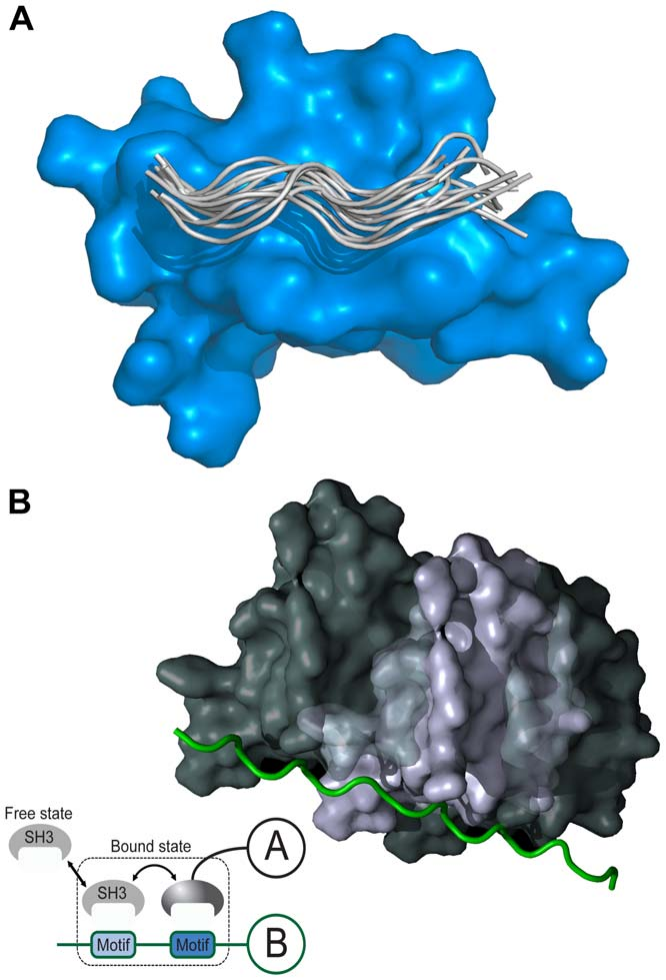
Cartoon representation of SH3 domain-ligand complex. (a) Multiple conformations of the peptides bound to an SH3 domain were collected from various crystal structures and aligned using MODELLER. The peptides form a polyproline II structure on the binding surface of an SH3 domain. (b) A physical concept involved in the MS model is shown, where Motifs denotes binding sites in a proline-rich region. An SH3 domain of a protein shuttles between each binding site on a proline-rich region of a partner protein.

Protein-binding modules mediate protein interactions [8]. The Src homology 3 (SH3) domain is one of the most abundant protein-binding modules and is shown in Figure 1a. More than 11,000 different SH3 domains can be retrieved from SMART's non-redundant database [9]. There are various consensus sequences for SH3-binding ligands, which are usually composed of fewer than 10 residues [10], [11], [12]. SH3 domains recognize proline-rich regions that are typically composed of a “PxxP” binding motif, and residues at the flanking sides of the motif determine the orientation and specificity of the binding interaction [13]. However, it has also been reported that SH3 domains bind peptide ligands that lack the PxxP motif [13], [14], [15].

SH3-mediated interaction is weak and transient. Many SH3 domains have micromolar affinity to their putative ligands [16]. Nck adaptor protein increases the binding affinity via cooperation with multiple SH3 domains [17]. Mutation studies have shown that the surface of the SH3 domain binding to a peptide ligand was not fully optimized by evolution to form a stable complex [18]. Using a fluorine-based NMR study, Evanics et al. reported that the Fyn SH3 domain, while 96% of them being in a bound state, had an average exchange rate of 5200 s^−1^ between the free and bound states [19]. Using a computer simulation of SH3 docking, Ahmad et al. reported that electrostatic effects enhanced encounter complex formation and stabilized the transient complex [4]. Moreover, certain SH3-binding proteins may have multiple binding sites as shown in Figure 1b. For example, the SH3 domain in amphiphysin (P49418) recognizes the “PxRPxR” binding motif; the Arg/Pro-rich region of the Itch protein (containing the “PSRPPRPSR” sequence) has two binding sites for the amphiphysin SH3 domain [20]. Thus, to describe SH3-mediated interaction, the properties of the encounter complex and its dynamic equilibrium have to be considered. However, existing computational models for the SH3 interaction ignore the dynamic nature of complex formation and assume a stable complex [21], [22], [23], [24].

In this study, we developed a Multiple-Site (MS) model, which was derived based on the formalism for the standard free energy of binding [25] and used for describing SH3-mediated interaction, in which an SH3 domain recognizes its partner protein through closely located multiple binding sites on the surface of the partner protein by transiently docking each binding site. In the model, each site binds to the SH3 domain with its individual binding free energy. To verify the analysis results for the individual binding free energies, we compared the free energies with those calculated using FoldX, which is a well-established algorithm based on atomistic force fields [26]. Additionally, we defined a parameter, called the maximum local population (MLP), as a metric to measure the contribution of a specific binding site that dominantly contributes to the complex population. To test this model, we used the positionally addressable synthesis of peptides on continuous cellulose membrane supports (SPOT-synthesis) experimental data of SH3 domains reported by Landgraf et al. [12], which provided semi-quantitative dissociation constants for SH3-peptide complexes [12], [27]. In this study, we show that our model better describes the data than an alternative conventional model by assuming that SH3 domains recognize their partners through closely located multiple binding sites. Finally, we discuss the physical basis for and biological meaning of the proposed computational model.

## Methods

### Protein-protein interaction via multiple binding sites

Protein complexes with weak binding affinities are in a dynamic equilibrium between the free and bound states. Previous studies have focused on describing the formation of a stable complex between proteins. However, in certain protein-protein interactions, a protein may bind at multiple binding sites on a partner protein. To depict the protein-protein interaction mediated by multiple binding sites, we derived an equation based on the formalism of the standard free energy of binding.

The standard free energy of binding depicts the binding phenomenon, in which two proteins form a complex with a nonbonding interaction [25]. Considering an equilibrium state in which the proteins *A* and *B* are dissolved in solvent, the standard binding free energy of the protein complex *AB* at equilibrium, *G*, is written as follows:



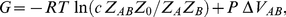
(1)where *P*, *c*, *R*, and *T* denote the standard pressure, a constant, the gas constant, and the temperature, respectively. *Z* denotes configuration integrals: *Z*_0_ is a configuration integral of the solvent molecules, and *Z_D_* is the configuration integral when a protein or protein complex *D* is dissolved in the solvent. Δ*V_AB_* is the volume change between the bound and free states of the complex of proteins *A* and *B*. The volume change causes work, but the pressure-volume work, *P* Δ*V_AB_*, is typically very small at standard pressure because Δ*V_AB_* is small [25].

We generalized the standard free energy formula by expanding the configuration integral of the protein complex, in which a protein *B* binds at multiple binding sites on a protein *A* by randomly shuttling between all of the sites. The configuration integral of the complex is written as follows:
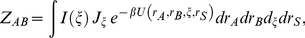
(2)where *ξ* denotes the coordinates of protein *B* relative to protein *A*, *J_ξ_* is the Jacobian determinant for the Eulerian rotation, *U* is the potential energy function of the molecular system, and *I*(*ξ*) denotes the binding criteria for the complex [25]. If the two proteins are sufficiently close together and measured as a bound state by a used experimental method, then *I*(*ξ*) is equal to 1; otherwise, this term is equal to zero. Generalization of the equation is achieved by assuming the criteria such that if protein *A* has various, well-separated binding sites for protein *B*, then *I*(*ξ*) is equal to 1 when protein *B* approaches each binding site of protein *A*; otherwise, this term is equal to zero. Simply, integration of *Z_AB_* is equal to zero in most regions of the coordinates *ξ* except for a few regions containing binding sites. If we label the regions as *B*1, *B*2, and so on, then the configuration integral is described by the linearly additive terms of the contributions of each binding site, as follows:
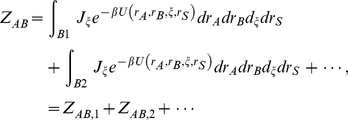
(3)where integrals are conducted in the regions; *Z_AB,i_* denotes a configuration integral of the protein-protein complex when protein *B* is localized at the *i*th binding site of protein *A*.

By combining Eqs.1 and 3, we derived the following equation:
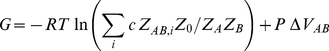
(4)To simplify the formula, we defined the individual binding free energy, in which protein *B* binds only at the *i*th binding site of protein *A*, as follows:
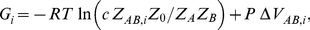
(5)where Δ*V_AB,i_* is the volume change between the bound and free states of the complex of proteins *A* and *B* when protein *B* binds to *i*th binding site of protein *A*.

By combining Eqs. 4 and 5, we derived a binding free energy of the proteins *A* and *B* as terms of individual binding free energies as follows:
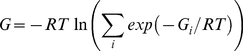
(6)where we assumed the individual volume changes are nearly equal to Δ*V_AB_*. Furthermore, dividing by *RT* made the binding free energy unitless. The final equation of the binding free energy is as follows:
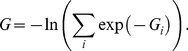
(7)where the binding free energies can be converted into the real binding free energies by multiplying by *RT*. This equation converges to the equation for the standard free energy of binding if only one binding site contributes to the binding interaction and explicitly includes the idea that additional binding sites near the best binding site will increase the population of the complex

### Experimental data preparation

SH3 domains bind at a proline-rich region that adopts a polyproline II structure on the binding surface of the SH3 domains and is composed of dozens of residues, as shown in Figure 1. Because the MS model requires large numbers of binding free energies, we used the binding free energy data from SPOT synthesis technology. SPOT synthesis has previously been used to screen peptides that bind to proteins, nucleic acids, and small ligands [28], [29]. Using the physical property of a correlation between the SPOT intensities and binding free energies [12], [27], a large amount of binding free energy data can be collected. We used the SPOT peptide array data reported by Landgraf et al. for preparing the binding energies for the SH3-ligand complex, where the data were given as SPOT intensities and peptide sequences [12]. The SPOT synthesis data were prepared using the following methods [12]. Phage display experiments were conducted to identify a consensus sequence for SH3-binding peptides. Based on this consensus sequence, the peptides for SPOT synthesis were collected by screening the yeast and human proteomes for their respective SH3 domains. These peptides were prepared with a longer sequence (13 or 14 amino acids) compared with the consensus (from 6 to 9 amino acids), where the longer sequences were selected from yeast and human proteomes. Thus, the sequences may contain multiple binding sites for SH3 domains. We used the SH3 domains from Abp1 (P15891), Myo5 (Q04439), Boi1 (P38041), Boi2 (P39969), Sho1 (P40073), Rvs167 (P39743), Lsb3 (P43603), Ysc84 (P32793), amphiphysin (P49418), and endophilin-1 (Q99962). We randomly selected 1,000 SPOT-synthesis data for each domain and used the negative natural logarithm of the SPOT intensity as the pseudo-binding free energy (-ln[BLU], where BLU [Boehringer light unit] is an arbitrary light intensity unit provided by the Lumi-Imager instrument).

### Multiple-Site (MS) model

**Figure 2:**
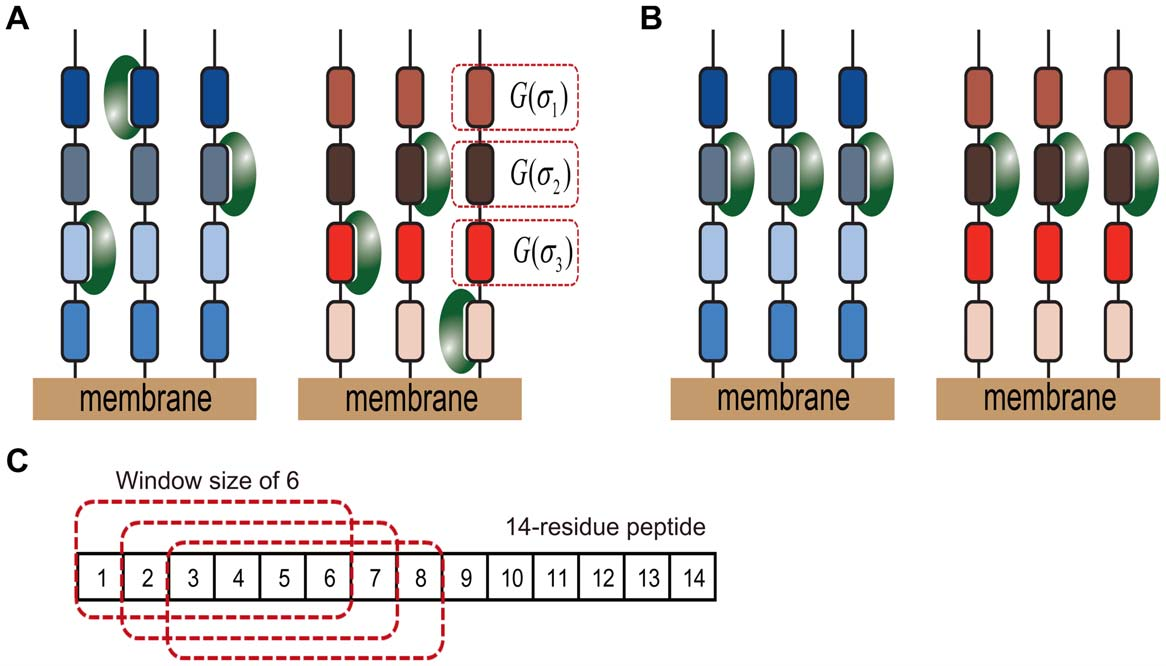
Two different binding models for SH3 complexes. (a) A graphical representation of the MS model, wherein short peptides with the same sequence were synthesized on a small region of membrane and SH3 domains are assumed to bind at various sites on the peptides. *G*(*σ_i_*) denotes the binding free energy of the *i*th binding site. An SH3 domain may bind at multiple sites on a single peptide, but temporally, one site per peptide due to the SH3 domain size; each site is in dynamic equilibrium with a different binding free energy. (b) A graphical representation of the SS model, which assumes that the SH3 domain binds at the same position on the peptides according to the alignment used in the SPOT experiment. (c) An example of multiple binding sites with a window size of 6 is illustrated for a 14-residue peptide, where the red squares with a dotted line denote individual binding sites and correspond to the individual binding sequences, *σ_i_* in Eq. 9.

The MS model was developed to describe the binding of a protein to its partner protein through closely located multiple binding sites on a surface of the partner protein by transiently docking at each binding site with an individual binding free energy. A graphical representation of the model for SH3-mediated interaction is shown in Figure 2a, in which an SH3 domain binds at multiple sites on the peptide that represents the SH3-binding region in a partner protein. To extract the individual binding free energy for each binding site, we used SPOT-synthesis data, which consisted of peptide sequences and their relative binding free energies. These binding free energies include the random noise involved in the experimental procedure. For example, an uncertain peptide density on the membrane, the purity of the synthesized peptides, the washing step, and the uncertainty involved in the signal detection procedure can all contribute to noise [27]. We introduced a statistical method to reduce the random error involved in the experimental data. This statistical method consisted of two steps.

In the first step, we converted the peptide sequences into binary-number sequences. The peptide sequences can be decomposed by the combination of an invariant term, single-residue terms, and higher-order terms, where the invariant term can be regarded as a reference sequence, single-residue term as a single mutation, and higher-order term as multiple mutations. Although, inclusion of the higher-order terms improves the accuracy of the descriptions of the peptide sequences, it also requires a large amount of data. Thus, it is necessary to cut the higher-order terms in a certain level based on the amount of experimental data available. In this study, we used only an invariant term and single-residue terms in the sequence conversion. The sequence conversion scheme was simple: we assigned 1 to the first position of a binary number sequence for designating a reference sequence and converted the residues in the peptide into binary numbers composed of 19 sequential elements (an amino acid in the reference sequence was not counted), where the binary numbers indicated that a given peptide had a specific amino acid at a specific residue position. Concisely, by introducing an equation, the sequence conversion and its relation to the binding free energies can be rigorously described. If we consider an *N*-residue peptide *σ* = [*a*_1_, …, *a_N_*], where *a_s_* represents the amino acid at the *s*th residue position, which binds to an SH3 domain and has a binding free energy *G*(*σ*). Further, assuming *M* amino-acid possibilities at each residue site, *a_s_* can take on values from 0 to *M*-1, where each index corresponds to a specific amino acid, and the index zero to a reference amino acid of the corresponding residue position. The binding free energy is described as follows:
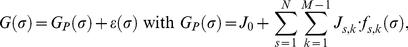
(8)where *G_P_*(*σ*) denotes a predicted binding free energy of a sequence *σ*. The *ε* term is an error, which is the difference between the binding free energy and its predicted value; the value is random and depends on the sequence. The *f_s,k_*(*σ*) term is the basis function of the sequence expansion and is equal to 1 if the site *s* in the sequence *σ* is occupied with the amino acid *k* and is otherwise zero. The *J* terms are the energies corresponding to the basis functions, where *J*_0_ and *J_s,k_* denote reference and single-mutation energies, respectively [30], [31]. From Eq.8, the problem of determining individual binding free energies is converted into finding the *J* terms.

In the second step, we combined Eqs.7 and 8 to derive an equation to fit the SPOT-synthesis data. The resulting equation for fitting is as follows:
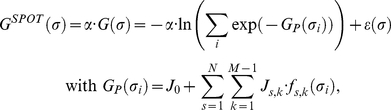
(9)where the binding free energy of the SH3-peptide complex is expressed by a combination of the contributions from multiple binding sites represented by the individual binding sequence *σ_i_* explained in Figure 2c. The inclusion of the parameter *α* improves the fitting accuracy by partially reducing errors in the conversion of SPOT intensities into pseudo-binding energies. In this equation, the pseudo-binding energies from SPOT-synthesis data *G^SPOT^* are used to find optimal *α* and *J* terms by reducing the fitting errors, *ε*. To determine these values, we used a MATLAB® script that was developed in-house (see Supplemental Information S1). Because the curve-fitting algorithm in the script would not give a global solution, we used several sets of initial *J* terms as inputs. Using the obtained *J* terms, the binding free energies of new sequences were further predicted.

### Single-Site (SS) model

The SS model was developed to simulate the binding phenomenon in which an SH3 domain is bound at a specific site of its partner protein. A graphical representation of the model is shown in Figure 2b, which depicts an SH3 domain that specifically binds at a site that represents a specific sequence region in a partner protein. For an *N*-residue peptide, there are *N-S*+1 possible binding sites in the peptide when the binding site consists of *S* residues as shown in Figure 2c. Because, in this model, we assume that each binding site is the only place to bind with an SH3 domain, there are *N*(*N*+1)/2 possible models for a training set.

To establish a relationship between the binding free energies and their sequences for each binding model, we converted the sequences of the binding sites into binary-number sequences, as explained in the MS model. Multiple linear regression analysis was applied to the binary-number sequences to minimize the fitting error, *ε*, in the following equation,
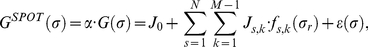
(10)where *σ_r_* denotes a representative binding site in the sequence *σ*, and the other parameters are explained in Eq.9. The equation for the binding free energy of the SS model is a special case of the MS model if only one binding site contributes to the binding interaction.

### Evaluation of prediction performance

We carried out a 10-fold cross validation using 1,000 randomly selected experimental data, which are listed in Supplemental Information S2. We processed the data by the following methods. First, 1,000 SPOT-synthesis data were equally assigned into 10 different data sets, of which each data set had 100 randomly selected SPOT-synthesis data. Second, 10 different [test, training] sets were prepared by circularly changing the role so that one data set was placed into a test set and the other 9 data sets into a training set. The training sets were used to derive the relationships between the peptide sequences and the binding free energies. Thus, 10 different training results were obtained for each SH3 domain. Each test set was used to assess the corresponding training results. We predicted the binding free energies of the peptide sequences and evaluated the root-mean-square (RMS) error between the predicted and the experimental values for each test set. As a result, 10 different RMS errors were obtained for each SH3 domain. Due to the small number of data in the test set, the RMS error depended on the standard deviation of binding energies. To remove the dependency, we divided each RMS error by the standard deviation value of a respective test set and named the resulting quantity “RMSE”. Thus, an RMSE of less than 1 indicates that the prediction is better than random; otherwise, the prediction is worse than random. Additionally, we used different *α* values for each SH3 domain in Eq.9, which were dependent on the training data. We used a median value among the *α* values for each SH3 domain. The selected *α* values were 12.3, 20.2, 28.7, 27.6, 2.7, 3.2, 5.9, 13.3, 5.0, and 3.6 for Boi1, Boi2, Abp1, Myo5, Sho1, Rvs167, Ysc84, endophilin-1, Lsb3, and amphiphysin, respectively. The selected *α* values were used to fit the training data again to assess the prediction performance for the test sets.

### Evaluation of the FoldX energies for the amphiphysin SH3 domain

We modeled the structure of the amphiphysin SH3 domain based on the crystal structure of the rat amphiphysin-2 SH3 domain (PDB entry 1bb9) using MODELLER [32], [33]. We used ten residues (AAPRRPPRAA) as an effective binding partner for the SH3 domain, of which we used the six core residues (PRRPPR) to simulate the complex binding and the alanines at the flanking sides for the conformational search that was irrelevant to the core binding [12], [23], [24]. To build a complex structure, we used the crystal structure of the C-Crk N-terminal SH3 domain complexed with the C3G peptide (PDB entry 1cka) as a template and built the structure using MODELLER [32], [34].

To sample stable complex structures, we carried out a molecular dynamics (MD) simulation of the modeled structure using the AMBER9 with AMBER 2003 force field [35]. The complex structure was neutralized using Na+ ions and solvated in 4,220 TIP3P water molecules. The particle mesh Ewald (PME) was employed to treat the long-range electrostatic interactions. The simulation was performed under the condition of 300 K temperature and 1 g/cm^3^ density. We performed 1 ns of simulation and collected complex conformations every 1 ps [36].

The conformations from the MD simulation were clustered using the clustering module in ROSETTA 3.2 [37]. From the clustering, 28 structures were selected as the templates for evaluating the binding energies. We obtained 15,135 independent sequences by the fragmentation of the peptides into six-residue peptides. The core residues in the structural templates were mutated to the six-residue peptides using the fixed-backbone design module in ROSETTA 3.2. The binding energies of the mutated structures were evaluated using FoldX. We used the minimum value among the binding energies for each sequence as the FoldX energy for the sequence.

### Maximum local population

To quantify the degree of binding specificity, we defined the maximum local population (MLP) which measures the maximum localization of a specific binding site as follows:
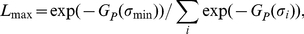
(11)where *L*_max_ denotes an MLP value and *σ*_min_ denotes a sequence with the minimum energy among the individual binding free energies. Thus, the MLP value represents the maximum percentage occupied by an SH3 domain at a specific site of a peptide.

## Results

Previous studies have reported that an SH3 domain forms a transient complex with other proteins [4], [19]. This physical phenomenon makes it difficult to measure the correct binding energy of the SH3 domain for a specific site of a protein because the domain may bind at other sites around the specific site. To overcome this difficulty, we developed a computational model based on a rigorous theoretical formula. This model facilitated the measurement of the correct binding energy and the determination of the underlying physics on the complex formation. We applied the model to the analysis of SPOT-synthesis data of various SH3 domains. In the process, we suggested two models: one model was used to fit the data under the condition of stable complex formation, and the other was used to fit the data under the condition of transient complex formation.

### Prediction of the binding free energies

It should be noted that the prediction performances of the proposed models depend on three factors: 1) the random error in the binding free energies, 2) the relationship between the sequences and the binding free energies (the primary source of unknown factors), and 3) a balance between the amount of data and the number of unknown factors. Because the random error depends solely on the experiment, computational improvement can be achieved by adjusting the remaining two factors.

### Multiple-Site (MS) model: Interaction mediated by multiple binding sites

In thermodynamics, a protein-protein interaction is described by an equilibrium state in which all possible binding conformations are considered to be adopted by the protein complex. Due to the weak and transient nature of SH3-mediated interactions, an SH3 domain may bind at multiple binding sites on a partner protein by shuttling among all of the sites in a short time. In the process of obtaining the SPOT-synthesis data, the binding sites of the partner proteins were selected by a consensus derived by a phage display technique, the peptide sequences of the selected binding sites were synthesized on a cellulose membrane, and the binding affinities to SH3 domains were measured by a spectroscopic method. Thus, the extracted peptide sequences, which were composed of fewer than 14 residues, represented the protein sequence. Because the consensus sequences for the SH3 domain-binding peptides were composed of fewer than 10 amino acids, the peptides could contain multiple binding sites for an SH3 domain. For an *N*-residue peptide, the number of binding sites is *N-S*+1 when an SH3 domain recognizes an *S*–residue peptide (called a window size of *S*), as shown in Figure 2c. Because the window size of SH3 domains is unknown, there are *N* possible models with different window sizes. In all of the models except the window size of *N*, the existence of multiple binding sites increases the population of the complex, as explained in Eq. 9.

**Figure 3:**
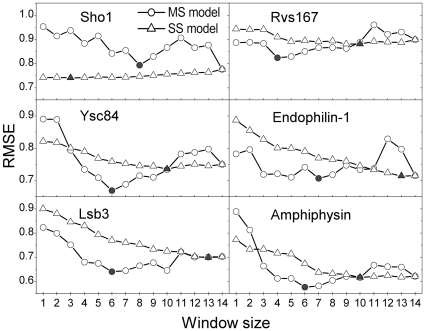
Comparison of the prediction errors between two computational models. The prediction errors from the MS and SS models are plotted as circles and triangles, respectively, and the points with the lowest prediction error are marked as solid circles and triangles. In this figure, the RMSE denotes the normalized prediction error against the experimental values.

In Figure 3, we show the results for 6 SH3 domains, where the balance between the amount of experimental data and the number of unknown factors determines the RMSEs. We plotted the RMSEs from the MS model according to window size as circles, and we particularly marked the data with the lowest RMSEs as solid circles. In this approach, a higher window size contains a greater number of *J* terms for fitting, which improves the prediction accuracy; however, the inclusion of more terms requires more experimental data because a small amount of data causes prediction bias. The RMSEs ranged from 0.5 to 1.1, and the best window size of the SH3 domains ranged from 4 to 8 residues (a window size of 4 for Rvs167; 6 for Lsb3, Ysc84, and amphiphysin; 7 for endophilin-1; and 8 for Sho1, where we excluded a full-length window size from the selection). These results are supported by a previous study, in which Cestra et al. reported that amphiphysin and endophilin-1 bind preferentially at 6- and 8-residue peptides, respectively, by analyzing phage display results [38].

#### Single-Site (SS) model: Interaction mediated by a single binding site

SH3 domains mediate protein-protein interactions that are implicated in various human diseases [39]. There have been various attempts to find inhibitors of SH3-mediated interactions for therapeutic purposes [39], [40]. In those studies, it was assumed that SH3 domains bind at one specific site of their partner protein. Based on this assumption, we devised the SS model, which considers such a condition in the fitting procedure.

In Figure 3, we represented the results of the SS model as triangles, and the optimal window sizes were marked as solid triangles, where each triangle for the window size of *S* designated the model which was selected among the *N*-*S*+1 possible models to have the minimum RMSE. As the window size increased, the RMSEs of all SH3 domains except Sho1 showed a monotonically decreasing pattern, indicating that almost all residues in the representative sequences contribute to the binding free energy. The reason for the increasing pattern of Sho1 is that one residue site in front of the “PxxP” motif dominantly reduced the RMSE value.

#### Comparison between models

We developed two computational models in the previous subsection, where the models adopted different physical binding phenomena to extract a relationship between the sequences at a binding site and the binding free energies. The SS model assumed that the complex was firmly bound, and it required the determination of both the window size and the correct binding site to find the best prediction model. The MS model assumed that the complex underwent dynamic binding, and it required the determination of the window size and the binding free energies of all binding sites. Thus, each model has a different number of unknown factors. The MS model requires a larger amount of data than the SS model because the MS model contains larger amounts of unknown factors to be determined.

As the window size increases, the models include more unknown factors to be determined. Because the amount of experimental data is fixed, the window size is the main factor that determines the prediction performance of the model. To compare the prediction performance between the two models, we divided the window sizes into three regions: from 1 to 2, from 3 to 9, and from 10 to 14. For the region between 1 and 2, the SS model had certain advantages because the peptide sequences that we analyzed were not selected randomly, in that specific amino acids at certain peptide positions were restricted. For example, if peptides contained the “PxxP” sequence motif, with fixed proline residues, then a window size of 2, which only varies the “xx”, was the same as a window size of 4 in the SS model, but this restriction had no effect on the MS model. This effect was observed in Ysc84 and amphiphysin, where the SS model using window sizes less than 2 outperformed the MS model using the same window size. However, both models performed similarly when using a window size of 3. In Sho1, we noted an extreme case of this restriction effect, where a window size of 1 already gave almost the best performance (the model with the window size of 1 has only 0.2% higher prediction error than the best model). For the region between 3 and 9, the MS model performed better than the SS model where the sequence-space-restriction effect disappeared. It was previously explained that improvements in prediction performance can be achieved by adjusting two factors. In this case, the better performance of the MS model in this window size region originated from the better description of the relationship between the sequences and the binding free energies because the other factor for better performance was unfavorable to the MS model. Lastly, for the region greater than 10, the SS model outperformed the MS model due to the smaller experimental data size.

**Figure 4:**
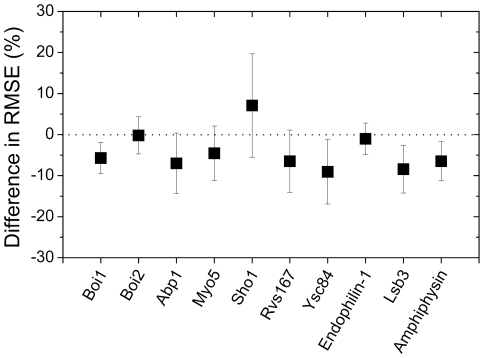
The prediction error difference between the MS model and the SS model. The differences in the prediction errors are plotted as squares, where a negative value denotes better performance of the MS model. The error bars denote the standard deviations of the differences for each SH3 domain. In this figure, the difference in prediction errors was measured by the following methods: the lowest prediction error was selected for each computational model, and the difference between the selected prediction errors from the models was evaluated for 10 training/test sets.

In Figure 4, the differences in prediction error between the models with the best performance are shown, where negative values indicate that the application of the MS model lowers the prediction error compared with the result from the SS model. All of the prediction errors were reduced except that of Sho1: 5.7-, 0.2-, 7.0-, 4.6-, 6.5-, 9.1-, 1.0-, 8.4-, and 6.5-percent decreases for Boi1, Boi2, Abp1, Myo5, Rvs167, Ysc84, endophilin-1, Lsb3, and amphiphysin, respectively. For Sho1, the SS model gave a 6.6-percent lower prediction error compared with the MS model. This improvement in the prediction performance indicated that the MS model provided a better method of describing the relationship between the sequences and the binding free energies.

### Dissociation constant

**Figure 5:**
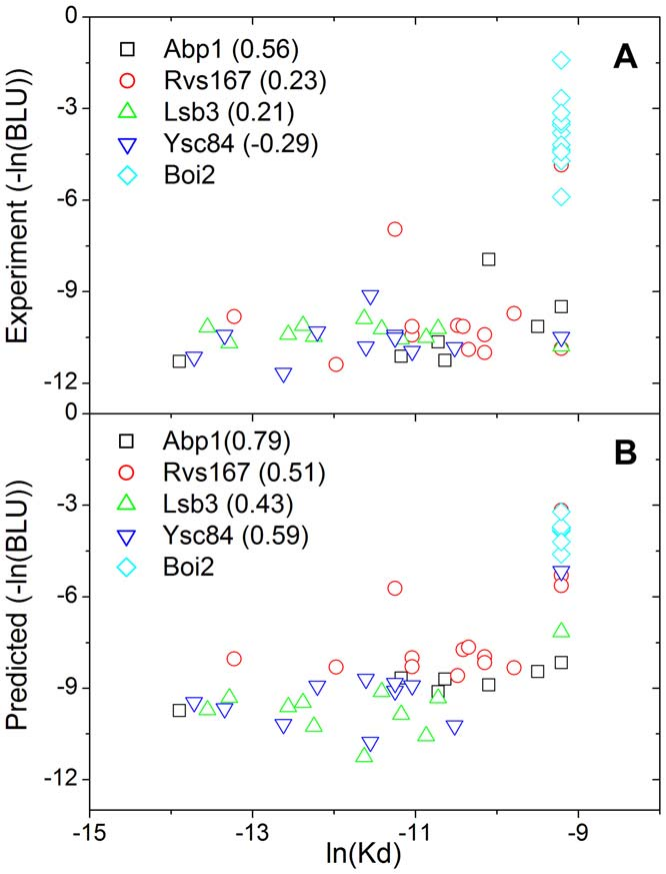
The MS model provides a better correlation with the dissociation constants than the prediction using the SPOT intensities. The predicted values for the SPOT intensities correlate better with the dissociation constants. (a) The experimental SPOT intensities were plotted according to their dissociation constants, and (b) their predicted SPOT intensities were plotted. The Pearson's correlation coefficient for each SH3 domain is in parentheses. The value for Boi2 is not provided because the dissociation constants are fixed due to experimental limitation.

Previous studies reported that SPOT intensities correlated with dissociation constants [12], [27]. However, the prediction of the dissociation constants directly using the SPOT intensities was hampered by the stochastic nature involved in the SPOT-synthesis experiment [27]. In Figure 5, the SPOT intensities are plotted according to their dissociation constants, where Pearson's correlation coefficients are 0.56, 0.23, 0.21, and −0.29 for Abp1, Rvs167, Lsb3, and Ysc84, respectively. Those correlations between the two experiments were improved by incorporating the predicted values from the MS model instead of directly using the pseudo-binding energies from the SPOT-synthesis experiment, where Pearson's correlation coefficients are 0.79, 0.51, 0.43, and 0.59 for Abp1, Rvs167, Lsb3, and Ysc84, respectively. This improvement in the correlation is related to the statistical averaging procedure contained in the model, which reduces the stochastic errors in the SPOT data [41], [42].

### FoldX energies

The MS model works by dividing the binding energy of a sequence into several binding energies of sequence fragments. To check the robustness of the MS model, we compared the binding energies of the fragments with the FoldX energies for the amphiphysin SH3 domain. Several experiments reported that the binding site of the amphiphysin SH3 domain was composed of 6-residues [24], [38]. We divided a 14-residue peptide sequence into nine 6-residue peptide sequences, labeled the nine sequences using the numbers from 1 to 9 on the basis of the starting position of the sequence, applied the procedure to all of the remaining 14-residue peptide sequences, grouped the 6-residue peptides into nine groups according to the labels, and calculated the Pearson's correlation coefficient between the FoldX energies and the pseudo-binding energies for each group. In Table 1, the direct comparison of FoldX energies with the pseudo-binding energies from the SPOT intensities gave Pearson's correlation coefficients from −0.09 to 0.34; the coefficient for 15,135 independent 6-residue peptides was 0.10. These lower correlation coefficients originated from the difficulty of identifying the correct sequences for the binding free energies, where a pseudo-binding energy represented the interaction energy of an SH3 domain with a 14-residue peptide, whereas the FoldX energy represented that with a 6-residue peptide. In contrast, the MS model gave the binding energies for the six-residue peptides, which made it possible to compare correctly with the FoldX energies. The Pearson's correlation coefficients between the FoldX energies and the energies from the MS model ranged from 0.16 to 0.62; the coefficient for 15,135 independent 6-residue peptides was 0.66.

**Table 1:** A comparison of FoldX energies with experimental values.

	Correlation coefficient								
INDEX++	1	2	3	4	5	6	7	8	9
**SPOT** *	0.13	0.24	0.14	−0.09	0.25	0.14	0.07	0.34	0.16
**MS Model** +	0.57	0.31	0.50	0.60	0.16	0.53	0.47	0.52	0.62

The Pearson's correlation coefficients between the FoldX energies and the experimental data are shown.

++ **INDEX** denotes a starting position in the 14-residue peptides to select representative fragment sequences for energy evaluation.

* **SPOT** denotes pseudo-binding energies.

+ **MS Model** denotes the binding free energies derived from the MS model (MS-model energies).

We used 2010 SPOT synthesis data for amphiphysin from reference [12]. We took six consecutive residues in peptide sequences to evaluate the FoldX and MS-model energies.

### Localization analysis of SH3 domains

**Figure 6:**
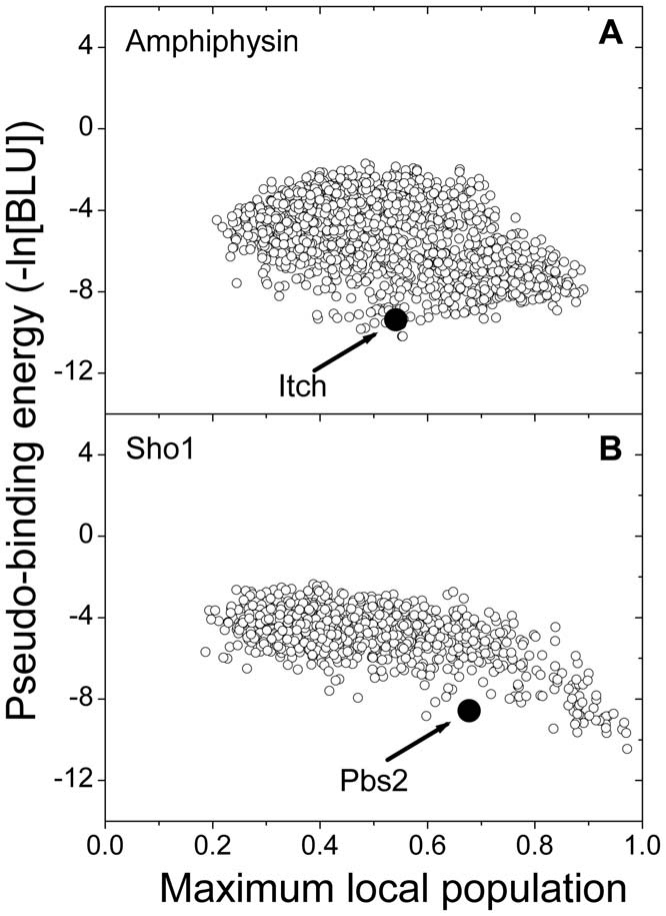
The maximum local populations for representative SH3 domains are shown. The predicted pseudo-binding energies are plotted based on the MLPs. (a) The MLP data for amphiphysin are shown. The maximum binding affinity data for amphiphysin fall within the middle region of the MLP. A solid circle marks the peptide (PSRPPRPSR) from the Itch protein that has two possible binding sites. (b) The MLP data for Sho1 are shown. The fully localized interaction for Sho1 has the maximum binding affinity. A solid circle marks the peptide (NKPLPPLPVAGSSKV) from the Pbs2 protein, which is a well-known binding partner of Sho1.

We evaluated the binding specificity of all of the peptides from the proteome using the MS model to show that an SH3 domain requires a different level of specificity, depending on its binding partners, for proper biological function. To quantify the degree of binding specificity, the MLP values were evaluated for SH3 domains, and two representative cases are shown in Figure 6. It is commonly shown that certain proteins prefer binding to an SH3 domain using a specific site (MLP larger than 0.7) and that other proteins prefer binding to an SH3 domain using multiple sites (MLP lower than 0.5). For amphiphysin, certain proteins have both lower pseudo-binding energies (approximately −10) and lower MLP values (approximately 0.5), which indicates that these proteins increase their binding affinity by providing multiple binding sites. It was verified by Western blotting that a proline-rich region of the ubiquitin ligase Itch, PSRPPRPSR, bound to amphiphysin [20]. Because the SH3 domain in amphiphysin recognizes the “PxRPxR” binding motif [38], the proline-rich region of the Itch protein has two binding sites for the amphiphysin SH3 domain. These multiple binding sites were also measured by the MLP value (approximately 0.5). For Sho1, a specific site in certain proteins bound with strong binding free energy (the MLP and pseudo-binding energy were approximately 1.0 and −11, respectively). The formation of a stable complex may be useful in certain biological functions, such as those of the binding partners of Sho1.

## Discussion

In this study, we proposed two different physical models to understand the binding phenomena for SH3 domains. The MS model, which assumes that multiple binding sites in a peptide contribute to SH3-mediated interaction, provides better results than the SS model, which assumes that only a single specific binding site contributes to binding interaction. The MS model displays improved performance because of the minute description of the binding complex. This description coincides with three observations. First, the binding sites of SH3 domains can dock with a limited number of amino acids. Second, SH3 domains have a weak binding affinity, which is in the micromolar range. Third, longer peptides have a stronger binding affinity with SH3 domains.

The SH3 domains, Rvs167, Lsb3, Ysc84, Sho1, endophilin-1, and amphiphysin, performed best when we used window sizes that ranged between 4 and 8. These window sizes were reasonable because they were similar to the number of amino acids in the consensus sequence. The MLP data indicated that the binding partners of an SH3 domain show different docking modes, providing a specific or multiple sites to the SH3 domain. For examples, the SH3 domain in amphiphysin binds to the proline-rich region in Itch with moderate specificity (MLP of 0.5) [20], whereas the SH3 domain in Sho1 binds to the proline-rich region in Pbs2 with high specificity (MLP of 0.7), where the complex formation is an important event for signaling in the high osmotic stress response pathway of yeast [43], [44]. Although Pbs2 has a high MLP and binding affinity, there are several proteins with higher MLPs and binding affinities than Pbs2. This result suggests that a strong binding energy does not ensure a proper biological function. As an example, Pbs2 has evolved to maintain a balance between protein complex stability and binding specificity for a biological function [44], [45]. The remaining SH3 domains, Abp1, Myo5, Boi1, and Boi2, performed best when we used window sizes less than 2. Although it is unclear whether those window sizes reflect the real binding properties, the MS model still improved the prediction performance compared with the conventional model.

The peptides that we used were longer than the consensus sequences because flanking residues were included [12]. These additional residues were useful for obtaining stronger SPOT signals and enabled us to collect a larger amount of data. However, this additional portion of the peptides generated obscure results, as the added portions provided additional binding sites. The MLP data show that many peptides bind to an SH3 domain using multiple sites. This observation introduces certain difficulties in the use of SPOT data intensities as a reference for computational modeling. For example, in other studies, SPOT data were used as the reference data, and a specific site on the peptides was used to represent the peptide region responsible for the binding free energy [23], [24]. These computational difficulties can be resolved using the MS model. Interestingly, for amphiphysin SH3 domain, the individual binding free energies were well correlated with the FoldX energies (Pearson's correlation coefficient of 0.66), and the ensemble of the individual free energies using the MS model also gave a good correlation with the pseudo-binding energies (Pearson's correlation coefficient of 0.82).

An analysis of the MLP data shows that the protein complex population grows by increasing either 1) the binding affinity between an SH3 domain and a specific site in a sequence or 2) the number of closely-located sites able to bind with an SH3 domain in a sequence. This difference in binding modes according to binding partners alludes that for achieving an appropriate biological function, proteins have evolved a part of their sequence which recognizes SH3 domains toward two different directions providing: 1) a specific sequence site for a specific biological function requiring a stable complex or 2) multiple sites to increase local population of the protein complex with preserving transient binding nature. Thus, the usage of multiple sites by proteins is expected to have various functional benefits, such as regulating protein localization without perturbing the dynamics of the complex, increasing the exchange rate of binding, and accelerating the speed of the complex formation.

In summary, we report a computational model that is designed to describe a protein complex bound with a weak and transient interaction. Next, we show that the application of this model improves the prediction performance for the binding free energies of SH3-peptide complexes, indicating that the model contains a more realistic description of the binding phenomenon than previous approaches. This observation provides a biological insight into the mechanisms by which certain proteins increase the local population around an SH3 domain by providing closely located multiple binding sites to the domain. This model also provides a new method of describing a weak and transient protein binding. Many proteins have a proline-rich region that is recognized by various domains with a weak and transient interaction, and these domains can be new targets for the application of the proposed model. One possible application is to search the entire proteome for binding partners because this model gives a better correlation between the predicted values and the dissociation constants. Future improvements can be achieved by incorporating other feature spaces, such as those used in the cluster expansion method [31], and alternative statistical methods that incorporate the proposed physical model for better prediction performance.

## Supporting Information

Supplemental Information S1 **Matlab script to fit the parameters in the MS model.**
(ZIP)

Supplemental Information S2 **List of selected peptide sequences for 10 SH3 domains.**
(XLS)
